# Protective Effects of Taurine Chloramine on Experimentally Induced Colitis: NFκB, STAT3, and Nrf2 as Potential Targets

**DOI:** 10.3390/antiox10030479

**Published:** 2021-03-18

**Authors:** Seong Hoon Kim, Hye-Won Yum, Seung Hyeon Kim, Wonki Kim, Su-Jung Kim, Chaekyun Kim, Kyeojin Kim, Young-Ger Suh, Young-Joon Surh

**Affiliations:** 1 Research Institute of Pharmaceutical Sciences, College of Pharmacy, Seoul National University, Seoul 08826, Korea; daduhoon@naver.com (S.H.K.); uknowyum@hanmail.net (H.-W.Y.); shjbeh@naver.com (S.H.K.); worldsadon@hanmail.net (W.K.); nynna79@snu.ac.kr (S.-J.K.); kyeojin01@gmail.com (K.K.); 2 Cancer Research Institute, Seoul National University, Seoul 03087, Korea; 3 Department of Pharmacology and Toxicology, College of Medicine, Inha University, Incheon 22212, Korea; chaekyun@inha.ac.kr; 4 College of Pharmacy and Institute of Pharmaceutical Sciences, CHA University, Seongnam 13488, Korea; ygsuh@snu.ac.kr; 5 Department of Molecular Medicine and Biopharmaceutical Sciences, Graduate School of Convergence Science and Technology, Seoul National University, Seoul 08826, Korea

**Keywords:** taurine, taurine chloramine, colitis, heme oxygenase-1, 2,4,6-trinitrobenzene sulfonic acid, NFκB, STAT3, Nrf2

## Abstract

Taurine chloramine (TauCl) is an endogenous anti-inflammatory substance which is derived from taurine, a semi-essential sulfur-containing β-amino acid found in some foods including meat, fish, eggs and milk. In general, TauCl as well as its parent compound taurine downregulates production of tissue-damaging proinflammatory mediators, such as chemokines and cytokines in many different types of cells. In the present study, we investigated the protective effects of TauCl on experimentally induced colon inflammation. Oral administration of TauCl protected against mouse colitis caused by 2,4,6-trinitrobenzene sulfonic acid (TNBS). TauCl administration attenuated apoptosis in the colonic mucosa of TNBS-treated mice. This was accompanied by reduced expression of an oxidative stress marker, 4-hydroxy-2-nonenal and proinflammatory molecules including tumor necrosis factor-α, interleukin-6 and cyclooxygenase-2 in mouse colon. TauCl also inhibited activation of NFκB and STAT3, two key transcription factors mediating proinflammatory signaling. Notably, the protective effect of TauCl on oxidative stress and inflammation in the colon of TNBS-treated mice was associated with elevated activation of Nrf2 and upregulation of its target genes encoding heme oxygenase-1, NAD(P)H:quinone oxidoreductase, glutamate cysteine ligase catalytic subunit, and glutathione *S*-transferase. Taken together, these results suggest that TauCl exerts the protective effect against colitis through upregulation of Nrf2-dependent cytoprotective gene expression while blocking the proinflammatory signaling mediated by NFκB and STAT3.

## 1. Introduction

Inflammatory bowel disease belongs to a distinct group of disorders characterized by prolonged systemic inflammation in the digestive tract. Crohn’s disease (CD), together with ulcerative colitis (UC), represents a prototypic form of inflammatory bowel disease (IBD). CD commonly affects the tail end of the small intestine (the ileum) and proximal colon while UC involves inflammation of the large intestine (colon) and the rectum [1]. The pathogenesis of CD as well as UC involves complex interactions among environmental factors, dysregulated immune response, gut microbiota, and disease susceptibility genes [2,3].

Chronic transmural inflammation of the intestinal wall provokes aberrant activation of the immune system. The hyper-reactive immune response often accompanies massive intracellular production of reactive oxygen species (ROS) with a concomitant decrease in the antioxidant defense. The resulting imbalance between ROS production and the antioxidant capacity causes oxidative stress [3]. Oxidative stress leads to mucosal layer damage and bacterial invasion, which in turn further stimulate the immune response. This amplifies a pathogenic cascade and exacerbates colonic inflammation [2]. While much effort has been directed at clinical trials to help CD, the disease is still incurable. 

2,4,6-Trinitrobenzene sulfonic acid (TNBS)-induced inflammation in mouse colon is one of the most commonly utilized animal models that replicate human CD [4,5,6]. In this model, intrarectal administration of TNBS produces symptoms similar to that of CD. These include bloody diarrhea, rectal bleeding, body weight loss, large bowel wall thickening, more diffuse intestinal inflammation, occasional adhesions, fibrosis, vascularized ulceration, etc. [5,6]. This experimental model has provided valuable insights to the molecular basis of the disease development and progression and also the preclinical testing of various anti-inflammatory and/or anti-oxidative compounds in regards to their potential to prevent the development or delay the progress of CD [5]. 

Despite remarkable progress in our understanding of the pathophysiology of CD, a cost effective and efficacious therapy has yet to be developed. Considering oxidative stress and inflammatory damage as prime etiological factors for CD, search for safe and easily accessible substances with pronounced antioxidant and anti-inflammatory activities can be a pragmatic approach in the management of the disease. Taurine, an amino sulfonic acid, is one such candidate. It is abundant in muscle meat (e.g., chicken and beef), fish (salmon) and eggs. Taurine has a wide spectrum of biological functions, such as conjugation of bile acids, antioxidation, osmoregulation, membrane stabilization, and modulation of calcium signaling [7]. 

Taurine reaches particularly high concentrations in some immune cells of inflamed tissues exposed to elevated levels of oxidants (e.g., neutrophils undergoing oxidative burst). This suggests that taurine may have a vital role in inflammation associated with oxidative stress. Indeed, at the site of inflammation, taurine reacts with and neutralize hypochlorous acid generated by the myeloperoxidase (MPO)-hydrogen peroxide (H_2_O_2_)-halide system of the neutrophils. This reaction results in the formation of less toxic, but biologically active taurine chloramine (TauCl) [8]. The antioxidative and anti-inflammatory properties of TauCl have been reported [9,10,11]. In this study, we investigated the effects of TauCl on TNBS-induced murine colitis and underlying molecular mechanisms.

## 2. Materials and Methods

### 2.1. Materials 

TauCl as a crystalline sodium salt (MW 181.57) was prepared as described previously [12]. Primary antibody for protein modified with 4-hydroxynonenal (4-HNE) was purchased from Japan Institute for the Control of Aging (JaICA), Nikken SEIL Co., Ltd. (Shizuoka, Japan). Primary antibodies for cyclooxygenase-2 (COX-2), Signal transducer and activator of transcription (STAT3), phospho-STAT3^Y705^, cyclin D1, α-tubulin, Kelch-like ECH-associated protein 1 (Keap1) and lamin B1 were provided by Cell Signaling Technology (Danvers, MA, USA), and those for Nrf2 and heme oxygenase-1 (HO-1) were supplied from Abcam (Cambridge, MA, USA) and Enzo Life Sciences (Farmingdale, NY, USA), respectively. Nuclear factor-κB (NFκB) p65 and phospho-NFκB p65 were obtained from Santa Cruz Biotechnology (Dallas, TX, USA). Antibodies for caspase-3 and cleaved caspase-3 were purchased from Cell Signaling Technology (Danvers, MA, USA).

### 2.2. Animals

Male C57BL/6 mice (5 to 6 weeks of age) were purchased from Orient Bio Inc. (Seongnam-si, South Korea). Mice were housed in plastic cages under controlled conditions of temperature (23 ± 2 °C), humidity (50 ± 10%) and light (12/12 h light/dark cycle). All animal experiments were complied with the Guide for the Care and Use of Laboratory Animals and approved by the Institutional Animal Care and Use Committee (IACUC) at Seoul National University (IACUC number; SNU-170823-2-2).

### 2.3. TNBS-Induced Colitis in Mice

Mice were anaesthetized with ketamine and xylazine. Colitis was induced by intrarectal administration of 2.5% TNBS (Sigma-Aldrich; St. Louis, MO, USA) in 50% ethanol via a thin round-tip needle equipped with an 1 mL syringe, and the animals were then kept in a vertical position for 30 s. The total injection volume was 100 μL. TauCl (20 mg/kg/day) was given by gavage for 10 days before induction of colitis and thereafter until the end of the study. The mice were euthanized 3 days after TNBS administration. The colons were removed, opened longitudinally and washed with phosphate-buffered saline (PBS). Immediately after washing, an inflamed segment was cut for histopathological examination, whereas another portion was flash frozen in liquid nitrogen and kept at −70 °C until use. 

### 2.4. Macroscopic Assessment

After 3 days of TNBS administration, the body weight of mice was measured every day. Disease activity index (DAI) was determined as the sum of scores of weight loss, rectal bleeding and stool consistency. Collected colon tissues were cut longitudinally, and the colon length was measured. 

### 2.5. Histology 

The mice were euthanized 3 days after TNBS administration. The isolated colons were washed with PBS. Immediately after washing, an inflamed segment was cut for histopathological examination, whereas another portion was flash frozen in liquid nitrogen and kept at −70 °C until use. Specimens of distal parts of the colon were fixed with 10% phosphate buffered formalin, and embedded in paraffin. Each section was stained with hematoxylin and eosin (H&E). The fixed sections were examined by light microscope (Nikon; Tokyo, Japan) for the presence of lesions. 

### 2.6. Measurement of MPO Activity

The MPO activity in mouse colon tissues was measured by using the Myeloperoxidase Colorimetric Activity Assay Kit (Sigma-Aldrich; St. Louis, MO, USA) according to the manufacturer’s instructions. 

### 2.7. Immunohistochemical Analysis

Immunohistochemical analysis of 4-HNE-modified protein was performed according to the standard protocols. Briefly, the freshly dissected colon tissues were fixed with 10% formalin, and the tissue block was embedded in paraffin. The paraffin-embedded tissues sections (5 μm thick) were then deparaffinised in xylene and transferred through graded ethanol for rehydration. Using microwave, the deparaffinized and rehydrated sections were heated twice for 6 min each in 10 mM citrate buffer (pH 6.0) for antigen retrieval. To extinguish endogenous peroxidase activity and diminish background staining, each section was treated with 3% hydrogen peroxide and 4% peptone casein blocking solution for 15 min. The slides were incubated with diluted primary antibody at room temperature for 40 min in Tris-HCl-buffered saline containing 0.05% Tween 20. After removing antibody solution and washing three times, the sections were incubated with horseradish peroxidase-conjugated mouse secondary antibody (Dako; Glostrup, Denmark). The tissues were treated with 3,3′-diaminobenzidine tetrahydrochloride substrate solution to reveal the color of stained antibody. Finally, counterstaining was carried out using Mayer’s hematoxylin.

### 2.8. Terminal Deoxynucleotidyl Transferase dUTP Nick End Labeling (TUNEL) Assay

Apoptotic DNA fragmentation was detected by the TUNEL assay with the ApopTag^®^ Peroxidase In Situ Apoptosis Detection Kit (Chemicon; Temecula, CA, USA). The isolated colon tissues were rinsed with PBS, and fixed in 10% buffered formalin (Sigma-Aldrich; St. Louis, MO, USA) for the TUNEL assay. The apoptotic cells were visualized by light microscope (Nikon; Tokyo, Japan). 

### 2.9. Immunofluorescence Staining

Colorectal specimens were fixed, paraffin-embedded and sectioned, and the sections were deparaffinized and rehydrated by serial washes with graded xylene and alcohol. For immunofluorescence staining, tissue sections were boiled in 10 mM sodium citrate (pH 6.0) for antigen retrieval, subjected to serial washing and permeabilized for 45 min at room temperature using 0.2% Triton X-100 in PBS and blocked with 3% bovine serum albumin (BSA) in PBS for 1 h at room temperature. The tissue sections were stained with primary antibodies for p65, p-p65, STAT3 and p-STAT3 (Cell Signaling Technology; Danvers, MA, USA) diluted at 1:250 in 3% BSA overnight at 4 °C. After washing three times each for 5 min to remove primary antibodies, tissues were incubated with appropriate secondary antibodies. Nuclei were counterstained with DAPI (Invitrogen; Carlsbad, CA, USA). Immunofluorescence images were collected with a fluorescence microscope (Nikon; Tokyo, Japan).

### 2.10. Tissue Lysis and Protein Extraction

Colon tissues were homogenized with the lysis buffer [20 mM Tris-HCl (pH 7.5), 150 mM NaCl, 1 mM Na_2_EDTA, 1 mM EGTA, 1% Triton, 2.5 mM sodium pyrophosphate, 1 mM β-glycerophosphate, 1 mM Na_3_VO_4_, 1 μg/mL leupeptin] including EDTA-free protease inhibitor cocktail tablet and 1 mM phenylmethyl sulfonylfluoride (PMSF)] in ice bath. The whole lysates were vortexed every 10 min for 3 h on the ice. The supernatants were collected and kept at −70 °C until use.

### 2.11. Preparation of Cytosolic and Nuclear Extracts

For preparation of cytosolic extraction, tissues were homogenized with buffer A [10 mM 4-(2-hydroxyethyl)-1-piperazineethanesulfonic acid (HEPES, pH 7.8), 1.5 mM MgCl_2_, 10 mM KCl, 0.5 mM dithiothreitol (DTT), 0.2 mM PMSF] followed by vortex mix every 10 min for 3 h in ice bath. The lysates were mixed with 10% Nonidet P-40 (NP-40) for 30 min before centrifugation. After centrifugation at 13,000× *g* for 15 min, the supernatants (the cytosolic extracts) were collected. Precipitated pellets were washed three times with buffer A containing 10% NP-40 to remove a residual cytosolic fraction. Nuclear pellets were resuspended in buffer C [20 mM HEPES, pH 7.8, 420 mM NaCl, 1.5 mM MgCl_2_, 0.2 mM EDTA, 0.5 mM DTT, 0.2 mM PMSF and 20% glycerol]. The nuclear lysates were vortexed every 10 min for 1 h, followed by centrifugation at 13,000× *g* for 15 min. The supernatants (nuclear extracts) were collected and stored at −70 °C until use.

### 2.12. Western Blot Analysis 

The total protein concentration was measured by using the Pierce^TM^ BCA Protein Assay Kit (Thermo Fisher Scientific; Rockford, IL, USA). Protein lysates (20 μg) were subjected to analysis by SDS-polyacrylamide gel electrophoresis (PAGE) and transferred to the polyvinylidene difluoride membrane (Gelman Laboratory; Ann Arbor, MI, USA). The blots were blocked with 5% non-fat dry milk in Tris-buffered saline containing 0.1% Tween 20 (TBST) for 1 h at room temperature. The membranes were incubated overnight at 4 °C with diluted primary antibodies. The blots were rinsed three times with TBST buffer for 10 min each. Washed blots were incubated with 1:5000 dilution of respective horseradish peroxidase-conjugated secondary antibodies (Invitrogen; Carlsbad, CA, USA) for 1 h and washed again three times with TBST buffer. The proteins were visualized with an enhanced chemiluminescence detection kit (Absignal) (Abclon; Seoul, South Korea) and LAS-4000 image reader (Fujifilm; Tokyo, Japan).

### 2.13. Reverse Transcription-Polymerase Chain Reaction Analysis (RT-PCR)

Total RNA was isolated from mouse colon tissues using TRIzol^®^ reagent (Invitrogen; Carlsbad, CA, USA) according to the manufacturer’s protocol. To synthesize the complementary DNA (cDNA), 1 μg of total RNA was reverse transcribed with murine leukemia virus reverse transcriptase (Promega; Madison, WI, USA) for 50 min at 42 °C and again for 15 min at 72 °C. About 1 μL of cDNA was amplified with a Solg^TM^ 2X Taq PCR Smart mix (SolGent Co., Ltd.; Daejeon, South Korea) in sequential reactions. The primers used for each RT-PCR reactions are as follows: *tnf-α,* 5′-TGA ACT TCG GGG GTG ATC GGT C-3′ and 5′-AGC CTT GTC CCT TGA AGA GAA-3′; *il-6*, 5′-AGT TGC CTT CTT GGG ACT GA-3′ and 5′-TCC ACG ATT TCC CAG AGA AC-3′; *cox-2*, 5′-CTG GTG CCT GGT CTG ATG ATG-3′ and 5′-GGC AAT GCG GTT CTG ATA CTG-3′; *actin*, 5′-AGA GCA TAG CCC TCG TAG AT-3′ and 5′-CCC AGA GCA AGA GAG GTA TC-3′; *ho-1*, 5′-TAC ACA TCC AAG CCG AGA AT-3′ and 5′-GTT CCT CTG TCA GCA TCA CC-3′; *nqo1*, 5′-AGG ATG GGA GGT ACT CGA ATC-3′ and 5′-AGG CGT CCT TCC TTA TAT GCT A-3′; *gclc*, 5′-GGC TAC TTC TGT ACT AGG AGA GC-3′ and 5′-TGC CGG ATG TTT CTT GTT AGA G-3′; *gss*, 5′-CCC ATT CAC GCT TTT CCC CT-3′ and 5′-GGG CAG TAT AGT CGT CCT TTT TG-3′; *nrf2*, 5′-CTT TAG TCA GCG ACA GAA GGA C-3′ and 5′-AGG CAT CTT GTT TGG GAA TGT G-3′ (forward and reverse, respectively). Amplified products were analyzed by 2% agarose gel electrophoresis, followed by staining with SYBR Green (Invitrogen; Carlsbad, CA, USA) and photographed using fluorescence in LAS-4000 (Fujifilm; Tokyo, Japan). 

### 2.14. Statistical Analysis

All the values were expressed as the mean ± standard deviation (SD) of at least three independent experiments. Statistical significance was determined by the Student’s *t*-test and *p* < 0.05 was considered to be statistically significant. All the statistical analyses were applied using GraphPad Prism 8.0 (GraphPad Software; San Diego, CA, USA). 

## 3. Results

### 3.1. TauCl Protects against TNBS-Induced Colitis in Mice 

**Figure 1:**
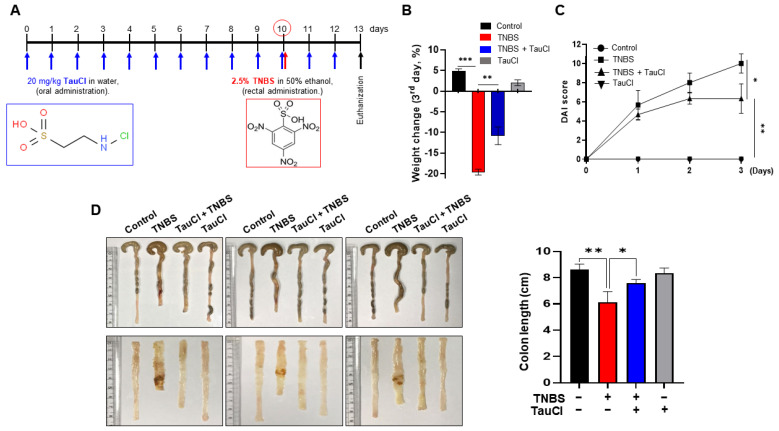
Effects of TauCl on inflammatory damage in the colon of TNBS-treated mice. (**A**) TauCl (20 mg/kg) was given on daily basis by gavage for 10 days before and for 3 days after intrarectal administration of 2.5% TNBS in EtOH. The comparison of body weight change (**B**), DAI (**C**), and colon length (**D**). Data are expressed as means ± SD (*n* = 3 for each group). *^,^ **^,^ *** Significantly different between groups compared (* *p* < 0.05; ** *p* < 0.01; *** *p* < 0.001).

**Figure 2:**
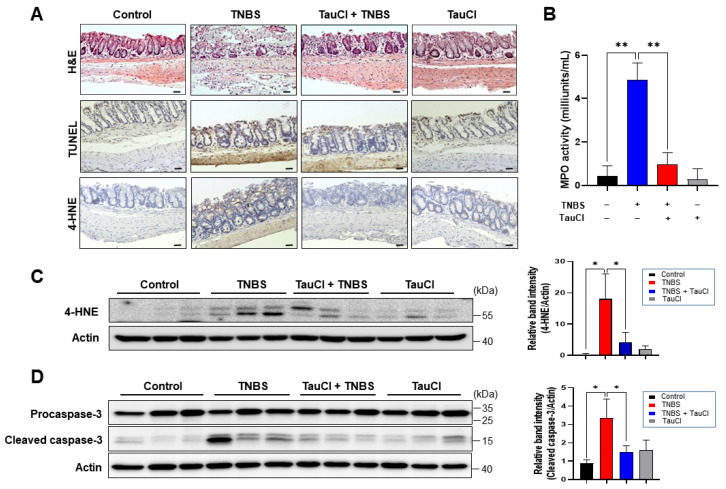
Attenuation of TNBS-induced colonic cell death, MPO activity, and oxidative damage by administration of TauCl. (**A**) Microscopic examination of H&E stained colonic mucosa from control mice and those treated with TNBS alone for 3 days, TNBS plus TauCl and TauCl alone. Immunohistochemical detection of TUNEL-positive cells and 4-HNE-modified proteins (brown spots) in mouse colon. Magnifications, ×100; Scale bar, 200 μm. (**B**) MPO activity in colonic mucosa. The enzyme assay was conducted as described in Materials and Methods. ** Significantly different between groups compared (** *p* < 0.01). (**C**) Western blot analysis of 4-HNE-modified protein expression in the colon strips of mice. Actin was used as an equal loading control. * *p* < 0.05. (**D**) Cleavage of caspase-3 was assessed by Western blot analysis. * *p* < 0.05.

Administration of TNBS (Figure 1A) via intra-rectal instillation caused severe body weight loss (Figure 1B), rectal bleeding and stool inconsistency (Figure 1C) and shortening of the colon (Figure 1D). All these abnormalities were ameliorated by administration of TauCl. Histopathological analysis showed that TNBS administration completely disrupted the architecture of colonic mucosa as evidenced by colitis exhibiting epithelial degeneration, crypt loss and inflammatory cell infiltration (Figure 2A). Administration of TauCl attenuated TNBS-induced mucosal damage of colon (Figure 2A). MPO is lysosomal enzyme abundant in neutrophils that are recruited to the inflamed site. The levels of MPO, as biomarkers of oxidative damage and inflammation, are often escalated in the inflammatory disorders, including IBD [13,14]. There was a robust increase in the colonic MPO activity in TNBS-treated mice, and this was dampened by TauCl administration (Figure 2B). 4-HNE is a reactive lipid peroxidation product that can modify protein. The TNBS-induced accumulation of 4-HNE-modified proteins in the colonic mucosa was attenuated by TauCl administration as assessed by immunohistochemical (Figure 2A) and immunoblot (Figure 2C) analyses. TauCl also protected against TNBS induced colonic cell death via apoptosis as assessed by immunohistochemical (Figure 2A) and Western blot (Figure 2D) analyses in terms of TUNEL staining and cleavage of caspase-3, respectively.

### 3.2. TauCl Administration Attenuates the Expression of Proinflammatory Cytokines and Enzymes in the Colon of TNBS-Treated Mice

**Figure 3:**
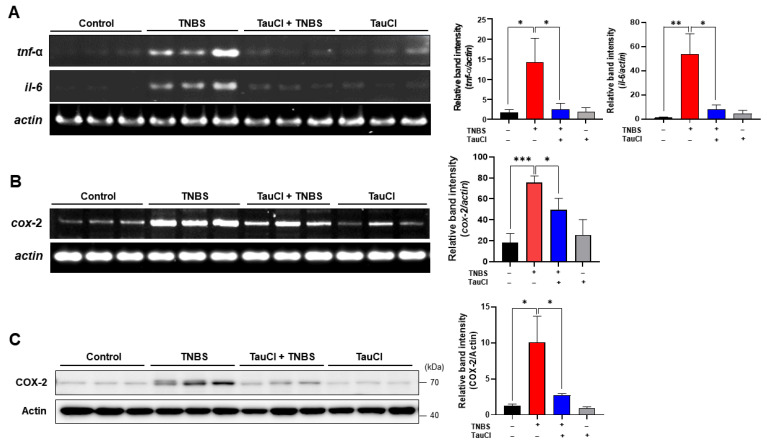
Effects of TauCl administration on expression of proinflammatory cytokines and COX-2 in mouse colonic mucosa. (**A**) RT-PCR analysis of mRNA expression of proinflammatory cyokines (*tnf-α*, *il-6*) and *cox-2*. (**B**,**C**) Colonic expression of *cox-2* and COX-2 expression was assessed by RT-PCR and Western blot analysis, respectively. Data are expressed as means ± SD (*n* = 3 for each group). *^,^ **^,^ *** Significantly different between groups compared (* *p* < 0.05; ** *p* < 0.01; *** *p* < 0.001).

**Figure 4:**
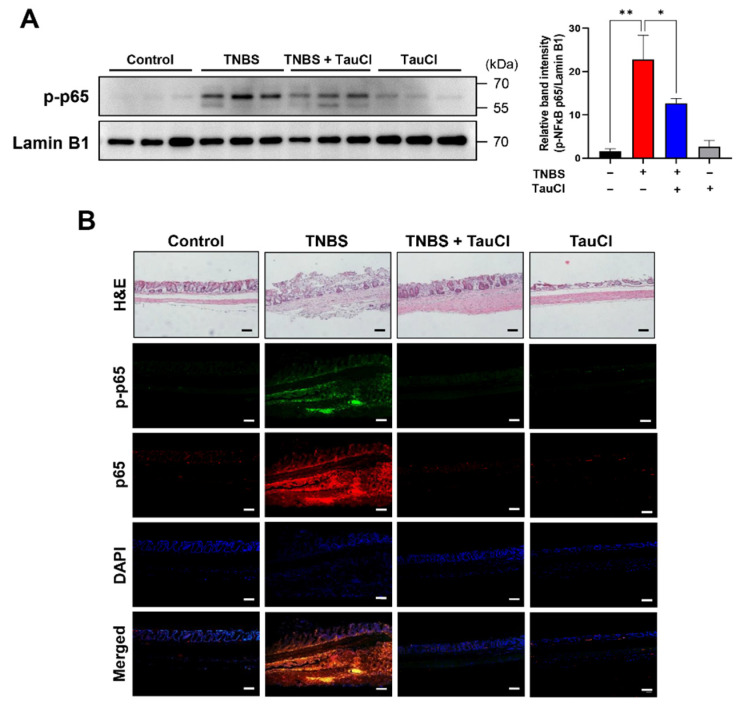
Inhibitory effects of TauCl on TNBS-induced p65 phosphorylation and nuclear localization of NFκB. (**A**) Expression of the phosphorylated NFκB p65 subunit was determined by Western blot analysis using nuclear extracts. (**B**) Accumulation of the phosphorylated as well as the total form of p65 was measured by immunofluorescence staining. The same tissue sections were stained with H&E. Scale bar, 200 μm. Results are presented as means ± SD. * *p* < 0.05; ** *p* < 0.01.

TNBS-induced colitis was accompanied by elevated colonic expression of genes encoding representative proinflammatory cytokines (TNF-α and IL-6) and enzymes (COX-2), which was suppressed by TauCl administration (Figure 3). Transcriptional regulation of these proinflammatory genes is mainly mediated by nuclear factor kappa B (NFκB). Under normal physiologic conditions, NFκB forms an inactive complex in the cytoplasm with the inhibitory protein IκBα. When cells are challenged with the inflammatory stimuli, IκBα underges phosphorylation which facilitates its proteasomal degradation via ubiqubiquitination. The degradation of IκBα allows release of NFκB for translocation into nucleus. p65 is a functionally active subunit of NFκB, and its phosphorylation is known to facilitate the nuclear translocation of NFκB and recruitment of coactivators, such as p300/CBP. As illustrated in Figure 4, there was pronounced nuclear localization of phosphorylated p65 (p-p65) in the colon of TNBS treated mice, and this was abrogated by TauCl administration.

### 3.3. TNBS-Induced Colonic STAT3 Phosphorylation Was Inhibited by TauCl Administration

**Figure 5:**
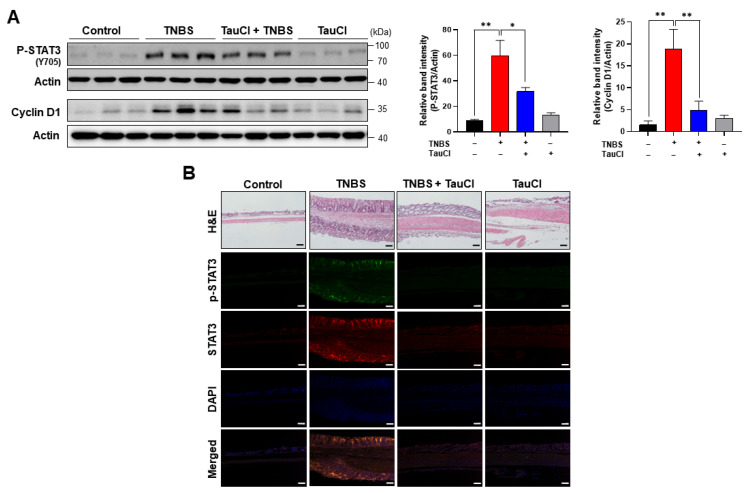
Inhibitory effects of TauCl on phosphorylation of STAT3 and its target protein expression in the colonic tissue of TNBS-treated mice. (**A**) Expression of P-STAT3 and cyclin D1 was measured by Western blot analysis. Actin was used as an equal loading control. *^,^ ** Significantly different between groups compared (* *p* < 0.05; ** *p* < 0.01). (**B**) Phosphorylated as well as total STAT3 was detected by immunofluorescence staining. The same tissue sections were stained with H&E. Scale bar, 200 μm.

Besides NFκB, STAT3 is another key player in inflammation and inflammation-associated carcinogenesis. One of the essential events in STAT3 activation is phosphorylation on tyrosine 705, which facilitates its nuclear translocation and transcriptional activity. TauCl administration significantly inhibited TNBS-induced phosphorylation of STAT3 and expression of its major target protein cyclin D1 (Figure 5). 

### 3.4. TauCl Administration Upregulates Antioxidant and Antiinflammatory Gene Expression

**Figure 6:**
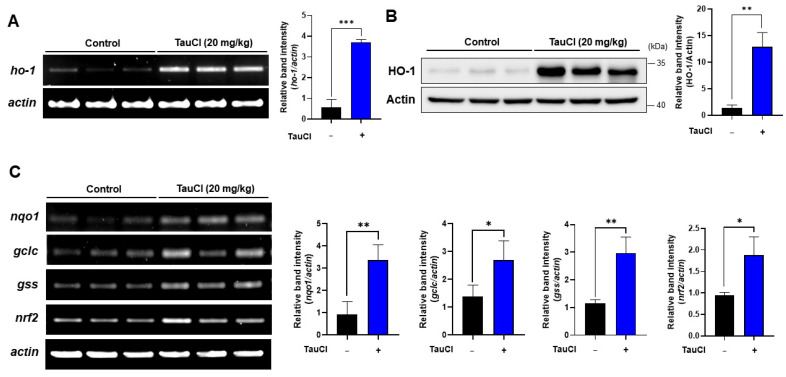
Upregulation of antioxidant signaling molecules induced by TauCl administration in mouse colon. The expression of *ho-1* (**A**) and its protein product (**B**) was determined by RT-PCR and Western blot analysis, respectively. (**C**) Expression of other antioxidant genes and *nrf2* was measured by RT-PCR. *^,^ **^,^ *** Significantly different between groups compared (* *p* < 0.05; ** *p* < 0.01; *** *p* < 0.001).

**Figure 7:**
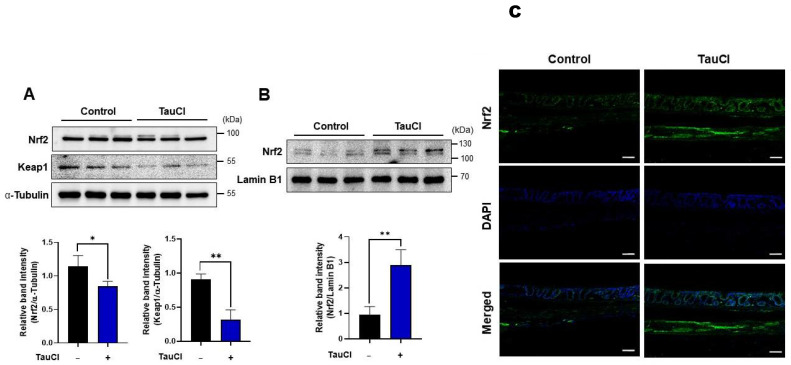
Induction of Keap1 degradation and nuclear translocation of Nrf2 by TauCl in mouse colon. Cytoplasmic levels of Nrf2 and Keap1 (**A**) and nuclear accumulation of Nrf2 (**B**) were measured by Western blot analysis. Nuclear localization of Nrf2 was verified by immunofluorescence staining (**C**). *^,^ ** Significantly different between groups compared (* *p* < 0.05; ** *p* < 0.01). Scale bar, 200 μm.

The cellular redox balance is maintained by a battery of antioxidant enzymes that counteract ROS. Many of these antioxidant enzymes also have anti-inflammatory functions. Administration of TauCl markedly enhanced the colonic expression of HO-1 at both transcriptional (Figure 6A) and translational levels (Figure 6B). Expression of genes encoding some other representative antioxidant/anti-inflammatory enzymes as well as Nrf2 was also upregulated in the colon of mice administered TauCl (Figure 6C). Nrf2 is a master switch in the transcriptional regulation of many antioxidant and anti-inflammatory genes. In unstressed conditions, Nrf2 is sequestered in the cytoplasm by Keap1 that degrades Nrf2 through ubiquitination. TauCl administration resulted in marked reduction in the cytoplasmic levels of Keap1 (Figure 7A) with concomitant increase in the nuclear accumulation of Nrf2 (Figure 7B,C).

## 4. Discussion

Taurine (2-aminoethylsulphonic acid), derived from the methionine and cysteine metabolism, plays an important role in various essential biological processes. Taurine is present at relatively high concentrations in neutrophils, granulocytes, lymphocytes and monocytes. Taurine is one of the most abundant free amino acids in our body with diverse biological functions [7]. The health beneficial effects of taurine have been generally attributable to its antioxidant and anti-inflammatory effects [8,15,16]. The tissue-protective potential of taurine has been assessed in various animal models of oxidative stress and/or inflammatory injuries. In an early study, oral administration of taurine by gavage following TNBS treatment ameliorated colitis and related symptoms in rats [17]. In another study, rats given taurine (1.5% *w*/*v*) in drinking water for 15 days before and 15 days after administration of TNBS solution developed less severe colitis and oxidative colonic mucosal damage. In addition, taurine reduced the expression of Bax and prevented the loss of Bcl-2 proteins in colonic mucosa of rats with TNBS-induced colitis [18]. Taurine supplementation significantly attenuated the weight decrease, diarrhea severity, colon shortening, and the increase in the colonic MPO activity in dextran sulfate sodium (DSS)-treated mice, another animal colitis model, mimicking clinical IBD. Taurine also significantly inhibited the increase in the expression of a proinflammatory chemokine, macrophage inflammatory protein 2 [19,20]. In a murine model of IBD-associated carcinogenesis, taurine administered in drinking water significantly suppressed azoxymethane plus DSS-induced colon carcinogenesis [21]. 

Neutrophils are predominant immune cells that protect the human body against pathogenic microbes and other harmful agents by eliminating them through phagocytosis. In fighting exogenous pathogens, neutrophils utilize one powerful weapon in their arsenal: the generation of the strong oxidant, hypochlorous acid (HOCl) which is nature’s germ killer [22]. HOCl is produced from H_2_O_2_ during the so-called oxidative burst by the MPO activity of the activated neutrophils in the presence of chloride ion. The escalation of MPO activity with concomitant generation of excessive HOCl, at the site of acute inflammation, can also be detrimental to host [22]. So it is necessary to detoxify or neutralize the residual excess HOCl as part of resolution of inflammation.

Taurine can act as a trap for HOCl forming the long-lived oxidant TauCl, which is more stable and less toxic than HOCl. Moreover, TauCl inhibits the generation of proinflammatory mediators by phagocytic cells [15]. So taurine exerts an anti-inflammatory as well as antioxidant action by preventing cytolytic damage caused by HOCl generated by inflammatory cells, particularly neutrophils. At the beginning of inflammation, neutrophils infiltrated into infected sites where they fight invaders and eventually die through apoptosis as a consequence of collateral host damage during oxidative burst. As the activated neutrophils undergo apoptosis, TauCl released into inflamed site stimulates the proresolving as well as anti-inflammatory action of macrophages [23,24]. We have previously reported that TauCl upregulates HO-1 expression in macrophages and thereby facilitates the engulfment of apoptotic neutrophils as well as pathogenic agents derived from infectious microbes [25,26,27].

Though taurine has been extensively investigated with regards to its anti-inflamma tory and antioxidative effects [8,9,10,28,29,30], there is paucity of data demonstrating the tissue-protective potential of TauCl in vivo. This may be partly due to the limitations on the availability of relatively large amounts of the compound. Based on the previous studies which evaluated the protective effects of taurine on experimentally induced colitis, we attempted to investigate the capability of its reactive metabolite, TauCl on TNBS-induced colonic inflammation in mice. Inflammatory cells, such as macrophages and neutrophils, secrete various cytokines and other signaling molecules that activate NFκB and STAT3 in epithelial cells. In the present study, oral administration of TauCl inhibited the activation of these two proinflammatory transcription factors, and expression of their respective target proteins, COX-2 and cyclin D1 in the colon of TNBS-treated mice. Besides anti-inflammatory activity, TauCl possesses antioxidant properties [8,9,10]. We have previously reported that TauCl can activate Nrf2, thereby upregulating the expression of HO-1, which account for its potentiation of macrophage-mediated phagocytic removal of apoptotic neutrophils during the resolution of acute inflammation [24,25]. We report here that TauCl activates Nrf2 signaling by inducing its nuclear translocation and upregulates its target protein HO-1 in the colonic mucosa. 

**Figure 8:**
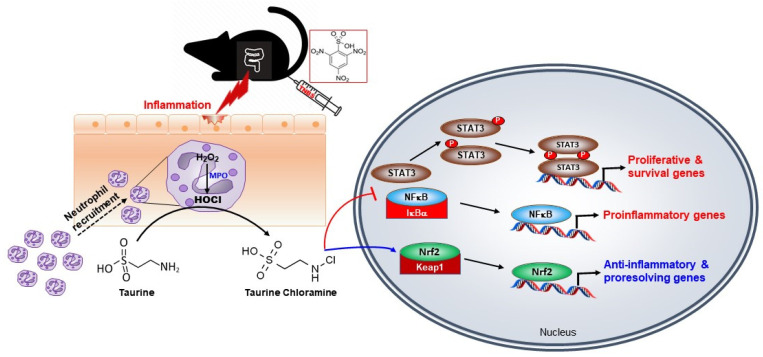
Molecular mechanisms by which TauCl protects against experimentally induced colitis. Abbreviations: MPO, myeloperoxidase; HOCl, hypochlorous acid.

Taken these findings all together, TauCl protects against experimentally induced colitis by inhibiting proinflammatory signaling while augmenting anti-inflammatory and possibly proresolving mechanisms (Figure 8). To the best of our knowledge, this is the first report on the protective effects of TauCl on experimentally induced colitis. Though TauCl is formed endogenously, its production is transient during inflammation and limited to some immune cells. Therefore, synthetic TauCl or its derivative in a more sustainable form may be considered for therapeutic application in IBD.

## Data Availability

Data presented in this study are included in the article.
